# Legacy of Hongxi Su: Pioneer of Chinese Cardiosurgery

**DOI:** 10.1007/s13238-021-00848-5

**Published:** 2021-08-18

**Authors:** Sumin Yang, Haoyu Hu, Xin Zheng, Shizhong Wang, Li Yuan

**Affiliations:** 1 Department of Cardiovascular Surgery, Affiliated Hospital of Qingdao University, Qingdao, 266001 China; 2 Department of Operation Room, Affiliated Hospital of Qingdao University, Qingdao, 266001 China; 3 Department of Anaesthesiology, Affiliated Hospital of Qingdao University, Qingdao, 266001 China

Prof. Hongxi Su (苏鸿熙) was born in Tongshan, Jiangsu Province, in January of 1915 (Fig. 1). He is the pioneer of Chinese cardiosurgery and one of the most renowned Chinese cardiothoracic surgeons, performing the first successful intracardiac surgery under extracorporeal circulation, as well as applying artificial blood vessels in aortic-carotid artery bypass surgery, shaping the cardiosurgery landscape of China.

**Figure 1:**
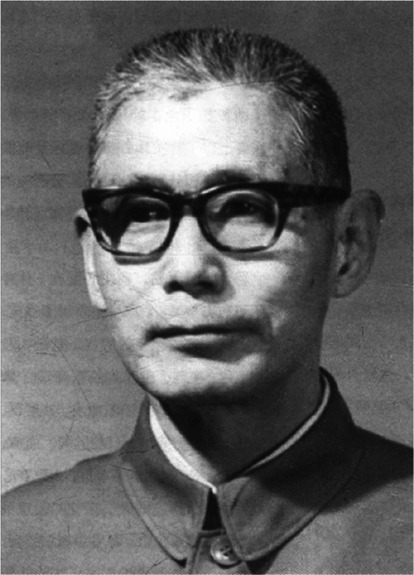
**Prof. Hongxi Su (苏鸿熙.** 2004**)**

In 1943, Prof. Su was appointed as an assistant at the School of Medicine of National Central University, and later promoted to the rank of instructor. In 1949, when the mainland had just been liberated, he received an invitation from Northwestern University’s Feinberg School of Medicine to study in the United States of America. With the support of Bocheng Liu (刘伯承), the mayor of Nanjing, he set out to America by cruise ship. During the trip, he wrote a poem:

A young man with graces appears on the deck.

He looks into the distance.

Geese are flying and circling.

The sea is endless.

Oh, my dear gentleman!

Where is your soul’s destination?

While studying in the United States, he succeeded in becoming a resident in surgery at Wissler hospital affiliated with Northwestern University’s School of Medicine and Chicago Tuberculosis Nursing Home. After that, he was admitted to the Cardiac Vascular Surgery of School of Medicine of the University of Illinois to pursue a graduate degree.

During this time, Dr. John Heysham Gibbon created a heart-lung machine, which was firstly applied in 1953, and enabled the first successful open-heart bypass surgery (Gibbon 1954). Prof. Su was aware of the significance of the heart-lung machine and seized the opportunity to observe animal experiments and clinical studies, aiming to gain insights into all aspects of extracorporeal circulation. He studied hard and spent the savings from many years to purchase two sets of the De Wall-Lillehei artificial heart-lung machine. At the time of China-United States tensions, Su’s family was prevented from returning, especially when they brought the machines with them. After careful arrangement, Prof. Su and his wife Mrs. Jin Su used tourist visas to separately head to London, where the machines had been delivered to. They went back to the motherland with two sets of artificial heart-lung machines via Canada, France, Czechoslovakia, and finally the Soviet Union.

## ADVANCING DESPITE DIFFICULTIES

After returning to China, Prof. Su worked in the First Affiliated Hospital of the Fourth Military Medical University. With the help of the headquarters and school leaders, Prof. Su established the Department of Cardiac Surgery. He even organized a research group concentrating on extracorporeal circulation and established an animal laboratory. The first animal experiment on extracorporeal circulation was carried out in June of 1957. Prof. Su led colleagues to study hemodynamics, pathophysiology, biochemical changes and myocardial preservation during extracorporeal circulation. With the progress of the studies, group members became increasingly skilled in extracorporeal circulation operation technology. They improved the equipment and established a set of management and work systems for the related operations. To improve the long-term survival rate of experimental animals after extracorporeal circulation operations, the group members performed careful animal experiments and postoperative nursing protocols, the same as operating on clinical patients. They observed the changes of experimental animals closely and dealt with them in time. At the same time, they summarized the experience and lessons of each experiment meticulously. The long-term survival rate of experimental animals increased to 76% in just half a year, laying a solid foundation for the safe transition to clinical practice (苏鸿熙, 1959).

**Figure 2:**
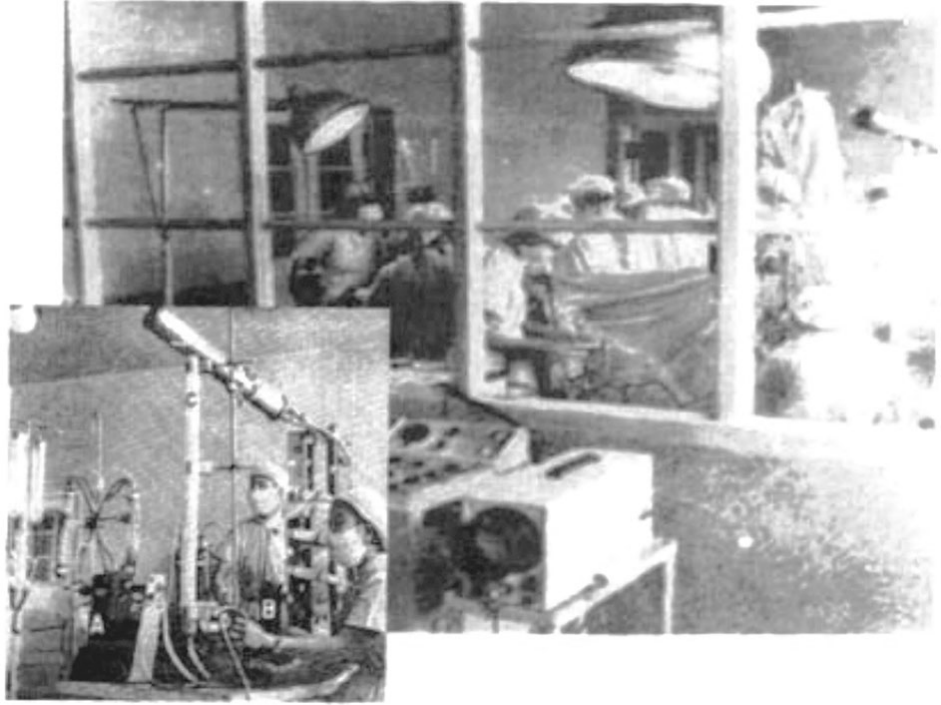
**The first intracardiac surgery under extracorporeal circulation in China**. The panel left below shows the artificial heart-lung machine in operation (刘维永, 2008)

Prof. Su carried out the first open-heart surgery under extracorporeal circulation in China with the aid of the Lillehei artificial heart and De Wall bubble oxygenator at 26th June 1958. The patient was a six-year-old child with a ventricular septal defect. The operation was successful, and the child recovered smoothly after surgery (苏鸿熙, 1959). The hospital followed the child for fifty years to track his growth and development, confirming that he lived a happy life and contributed to society. In 1963, Prof. Su performed the first successful application of a vascular prosthesis for aortic-carotid artery bypass grafting in China (Fig. 2).

After that, Prof. Su performed a series of studies on myocardial preservation during intracardiac operations under direct vision. He gradually constructed comprehensive theories and a series of protection methods for myocardial preservation, in addition to proposing the structural requirements of the artificial heart-lung machine, the rules of potassium metabolism before and after cardiopulmonary bypass together with a method of potassium supplementation (王爱华, 1983). Even more importantly, he firstly elucidated the mechanisms of intracranial hemorrhage and hematoma complicated by open heart surgery and put forward effective preventive measures (苏鸿熙, 1981).

Cardiovascular surgery had reached a turning point during the early 1950s, and cardiopulmonary bypass was the basic condition to carry out open heart surgery, facilitating meticulous and reliable cardiac operations under direct vision. Accordingly, cardiopulmonary bypass made it possible to cure many kinds of cardiovascular diseases. Prof. Su’s cardiac surgery under direct vision was one of the few successful clinical operations using a cardiopulmonary bypass in the world, occupying a very important position in the history of cardiac surgery (刘维永, 2008).

## SUCCESS DRIVEN BY LOVE AND CARE FOR PATIENTS

In 1972, Prof. Su transferred to the department of surgery, PLA General Hospital. “I always believe that no matter how famous, the doctor must be meticulous in the diagnosis and treatment of patients. Every complaint and every sign of the patient must be addressed. This is the science that must be followed in diagnosis. Any new project should be based on large amounts of literature and a full discussion of the state-of-the-art in the field (苏鸿熙, 1979). I often emphasize that the comrades in the department should go through the four stages of operations including preoperative diagnosis, emergency treatment, surgical treatment and postoperative care. For decades, I have adhered to the doctrine of staying with the postoperative patient until medically stable, not afraid of hardship and fatigue. When I worked at the Fourth Military Medical University, there was a case of postoperative blood pressure instability in a constricted cardiovascular patient, and I took care of him for seven days and nights. Finally, the patient recovered. While working in surgery and cardiac surgery, we treated a number of critically ill patients, for whom surgical treatment was very risky because of particularly complex conditions, and many patients being critically ill with heart failure. In this case, the surgeon needs to take responsibility and risk, and after long-term drug treatment, patients showed clinical improvement and became suitable for surgery. Finally, most patients recovered and could be discharged, while only few could not be saved. We should learn lessons from failures and get ready to move on.”

## BUILDING THE DEPARTMENT WITH STRATEGIC THOUGHT

In order to construct a solid foundation of reserve personnel, Prof. Su devoted himself to improving the professional quality of practitioners with strategic thought. He took an active part in academic work, holding a concurrent post as the first chairman of the Chinese Society for Thoracic and Cardiovascular Surgery and chief editor of *Chinese Journal of Thoracic and Cardiovascular Surgery*. He was also the chief editor of the publications *Modern Treatment of Multiple Injuries* and *Intensive Care Medicine*. In addition, Prof. Su participated in the publication of a series of professional books, including *Advances in Cardiovascular Surgery*, *Cardiovascular Surgery*, *Thoracic and Cardiac Surgery*, *International Thoracic and Cardiac Surgery* and so on. During his work at the Chinese Society for Thoracic and Cardiovascular Surgery, he was responsible for organizing three large-scale international conferences on thoracic and cardiovascular surgery and extracorporeal circulation, whereby he also conducted extensive academic exchanges. With Prof. Su’s great efforts, a large number of physicians were well trained and gained advanced knowledge from foreign counterparts by opening efficient channels for international exchange and cooperation. Generations of Chinese cardiac surgeons benefited from Prof. Su’s contributions for a long time.

## FAMILY, THE FIRMEST BACKING

Prof. Su’s success was greatly dependent on the support of his wife, Mrs. Jin Su. Their love lasted 62 years. Mrs. Su, formerly Jane, was an American. She married Prof. Su in the United States and went back to China with her husband without hesitation. In their late years, Prof. Su asked his wife, “Dear Jane, have you ever regretted coming to China with me?” Ms. Su answered in Chinese “我从来没有后悔过,我一直很高兴, 我要一辈子跟你就很高兴. (I never had any regrets. I’m always happy, just for spending my whole life with you.)”

“In those years, many accomplished overseas students gave up returning to China, mostly because of their wives being against it. My American wife Jane however, not only supported my return to China, but also offered me plenty of help in my career. It has been the honor of my lifetime.” Prof. Su said these words proudly at a gathering of returning friends many years ago.

Prof. Su was a staunch communist. He earned respect through trials and tribulations for many years, remaining an active member of the Communist Party of China until 99 years old.

Prof. Su passed away at 4:55 on July 31, 2018. He died peacefully without any invasive rescue measures according to his and his family’s will.

When the old man died, his legend became eternal.

May he rest in peace.
